# Medical Items

**Published:** 1897-01

**Authors:** 


### MEDICAL ITEMS.

---

A Happy New Year to all our readers, and, in the words of Tiny Tim, may God be with you, every one!

---

The New Orleans University Medical School is preparing to establish a training school for colored nurses. The movement deserves to succeed.

---

Perhaps a Misprint.—The local paper, speaking of a street accident, said: "Dr. Jones was quickly summoned, who, after examining the patient's purse, decided that the case was hopeless."—*Ex.*

---

Following are the officers of the Atlanta Society of Medicine for 1897: President, Dr. George H. Noble; Vice-President, Dr. L. H. Jones; Secretary, Dr. L. H. Hancock; Treasurer, Dr. L. B. Grandy.

---

The New York Polyclinic building was completely destroyed by fire on the morning of December 25th. Arrangements will doubtless be made for continuing the lectures and clinics in other quarters.

---

The Cleveland (Ohio) *Medical Gazette* comes out for November enlarged and greatly improved. We congratulate our valued contemporary upon beginning its twelfth volume with such evidences of good health and prosperity. May they never grow less.

---

Any of our readers who may desire an up-to-date cautery for office work will do well to write to the Carter Manufacturing Company, Louisville. Their Transformers for the electric light current are things of beauty as well as of service. See advertisement.

DR. C. DUNBAR ROY, of Atlanta, and Miss Carrie Ellett, of Richmond, were married December 16th. After their bridal journey in the North the happy couple returned to Atlanta, December 28th. THE JOURNAL offers best wishes for a long and happy life.

---

WE recognize in the last *Canadian Practitioner* two of our late editorials without any acknowledgment for the same. We are pleased to have our brother *Practitioner* use our matter, and only ask that proper credit be made for it. Don't let this happen again, brother.

---

A CODE of medical ethics three hundred years ago: "To travaille in this our vocation and arte, truely, rightly and without deceit, so that it may be to the glory of God, to the common welth, and our further knowledge, and finally to the health and safeguard of the people."—*Thomas Gale.*

---

THE *American Medico-Surgical Bulletin,* after January 1st, will be issued as a semi-monthly. The late editor, Dr. W. H. Porter, has been succeeded by Dr. R. G. Eccles. The *Bulletin* has come to be one of the best of our exchanges and a popular journal with physicians. We wish it continued success.

---

AT a surgical clinic a few days ago, before a class in the Harvard Medical School, a patient was shown who had a wound on the thigh caused by the bite of a rat. The instructor having asked the class for a diagnosis of the case, one of the students replied promptly, "rodent ulcer."—*Boston Med. and Surg. Journal.*

---

NIGHT-WORK is a much exaggerated evil of the physician's life. In the first few years of city practice there is not a superabundance of either day or night calls, and to one who falls asleep full of apprehensions as to the success of the future, the jingle of the telephone breaks in upon his troubled dreams like sweet music.—A. L. BENEDICT, in December *Lippincott's*.

---

Mrs. Goldstein (to ill Mr. G.): Isaac, der doctor say dot if you vould be cheerful und haf some gonfidence in him, dot vould be half der cure.

Mr. G.: Vell, if I do dot, vould he make a fifty per cent. reduction in der bill?—*Puck*.

---

THE Ware County (Georgia) Medical Society was organized last month at Waycross by Drs. J. L. Walker, J. L. Dedge, N. C. Berry, J. B. Bagley, G. P. Folks, T. J. Fales, J. J. Jones, A. P. English, N. A. Frier, R. P. Izlar. The following officers were elected: J. L. Walker, president; J. J. Jones, vice-president; A. P. English, treasurer; R. P. Izlar, secretary. We will be pleased to have the society make use of our pages for their proceedings.

---

R. M. W., in *Journal of American Medical Association*, wants to know the author of the following lines:

"God and the doctor we alike adore,
But only when in trouble, not before.
The trouble o'er, both are alike requited;
God is forgotten and the doctor slighted."

We pass.—*Texas Med. Journal*.

We turn it down.

---

A JOURNAL that gladdens the heart and delights the understanding every month is the *Texas Medical*, otherwise known as the *Redback*. As Chimmie Fadden would say, *it is up to de limit*. Standing for the ethical in both medicine and journalism, it is the uncompromising foe to the medical pretender and adventurer. It is a joy to its friends and a terror to its enemies; *suaviter in modo, fortiter in re*. There is but one *Texas Medical Journal*, and Brother

Daniel is the editor thereof. May its vigor never perish nor its complexion fade!

---

MR. W. B. SAUNDERS, of Philadelphia, the well known publisher of standard medical works, has in press a large work entitled "Anomalies and Curiosities of Medicine," by Dr. George M. Gould and Dr. Walter L. Pyle. It is an encyclopedic collection of rare and extraordinary cases, and of the most striking instances of abnormality in all branches of medicine and surgery, derived from an exhaustive research of medical literature from its origin to the present day. It will form a handsome octavo volume of 968 pages, with 295 illustrations in the text and twelve half-tone and colored plates. Ready in January.

